# Supplementary material for: Prostate Cancer Survivors Present Long-Term, Residual Systemic Immune Alterations
Source: Cancers (Basel). 2022 Jun 22;14(13):3058. doi: 10.3390/cancers14133058 (PMC9264868; doi:10.3390/cancers14133058)
Supplement: Supplementary file 1 [file cancers-14-03058-s001.zip › cancers-1734581-supplementary/Supplementary Figures, Tables - final merged.pdf]

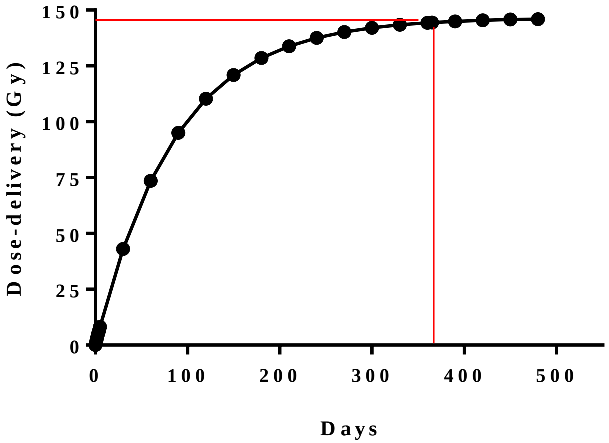

Supplementary Figure S1. Dose-delivery depending on treatment time of I^{125} isotope. Red line shows the total dose (145 Gy) 365 days after the seed implantation.

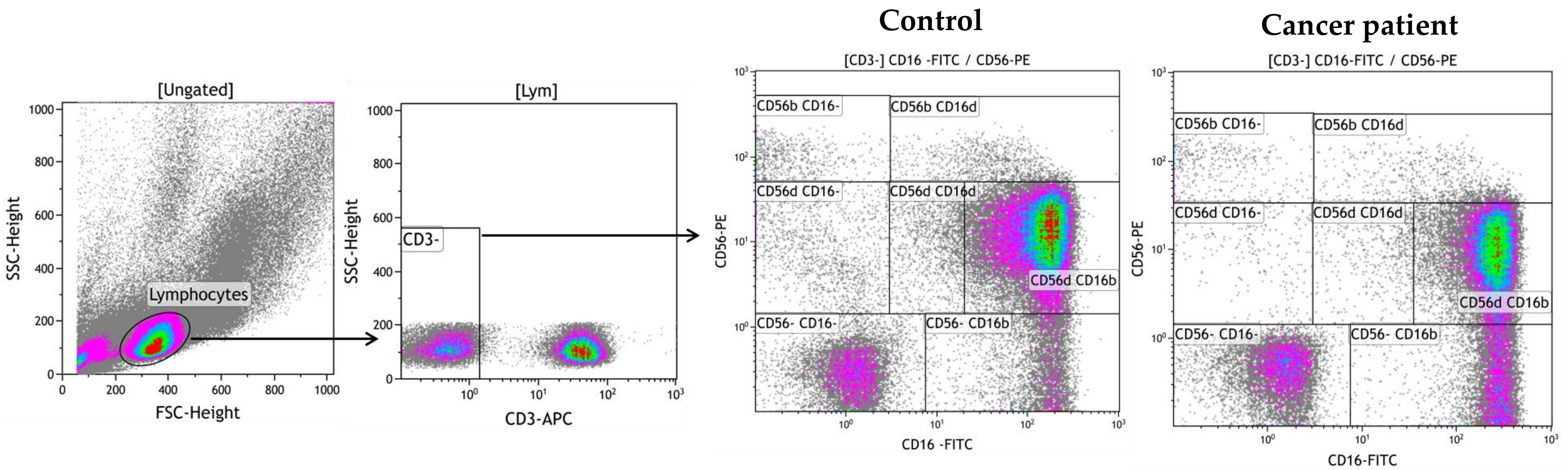

Supplementary Figure S2. Gating strategy of NK cell subpopulations and representative dot plots of a healthy control and a prostate cancer patient after radiotherapy. Five different maturation states of circulating NK cells were distinguished based on the expression level of the adhesion molecule CD56 and the activating receptor CD16 within the CD3- lymphocyte gate. These are immature precursor NK cells ($CD56^{bright}CD16-$), immature/early mature ($CD56^{bright}CD16^{dim}$), mature cytotoxic ($CD56^{dim}CD16^{bright}$), degranulating ($CD56^{dim}CD16-$) and anergic NK cells (CD56-$CD16^{bright}$) (Amand et al., 2017). Abbreviations: b: bright, d: dim

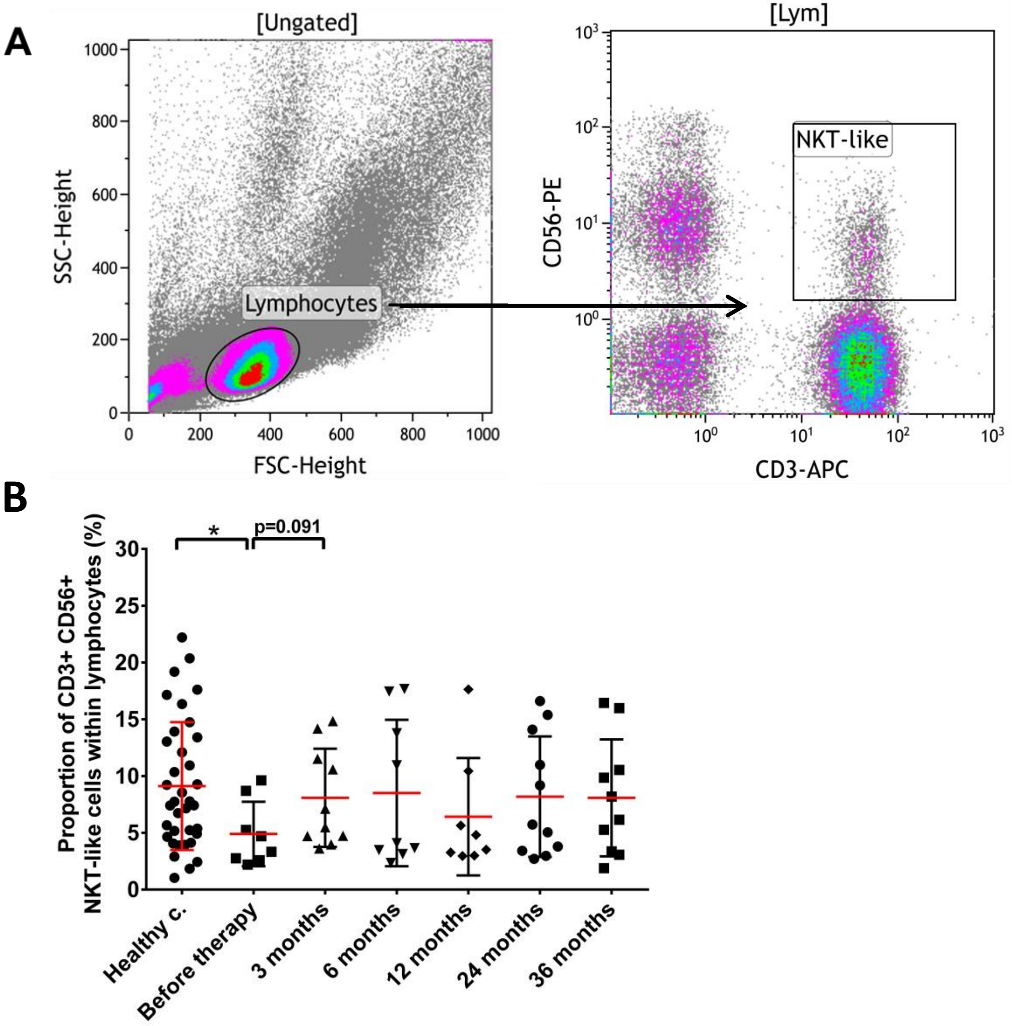

Supplementary Figure S3. Decreased NKT-like cell levels were detected in prostate cancer patients before treatment. (A) The gating strategy of CD3+CD56+ natural killer T (NKT)-like lymphocyte population. (B) Fraction of NKT-like cells. *n* (healthy controls) = 36, *n* (cancer patients) = 10-18. Significant differences were indicated with * ($p < 0.05$).

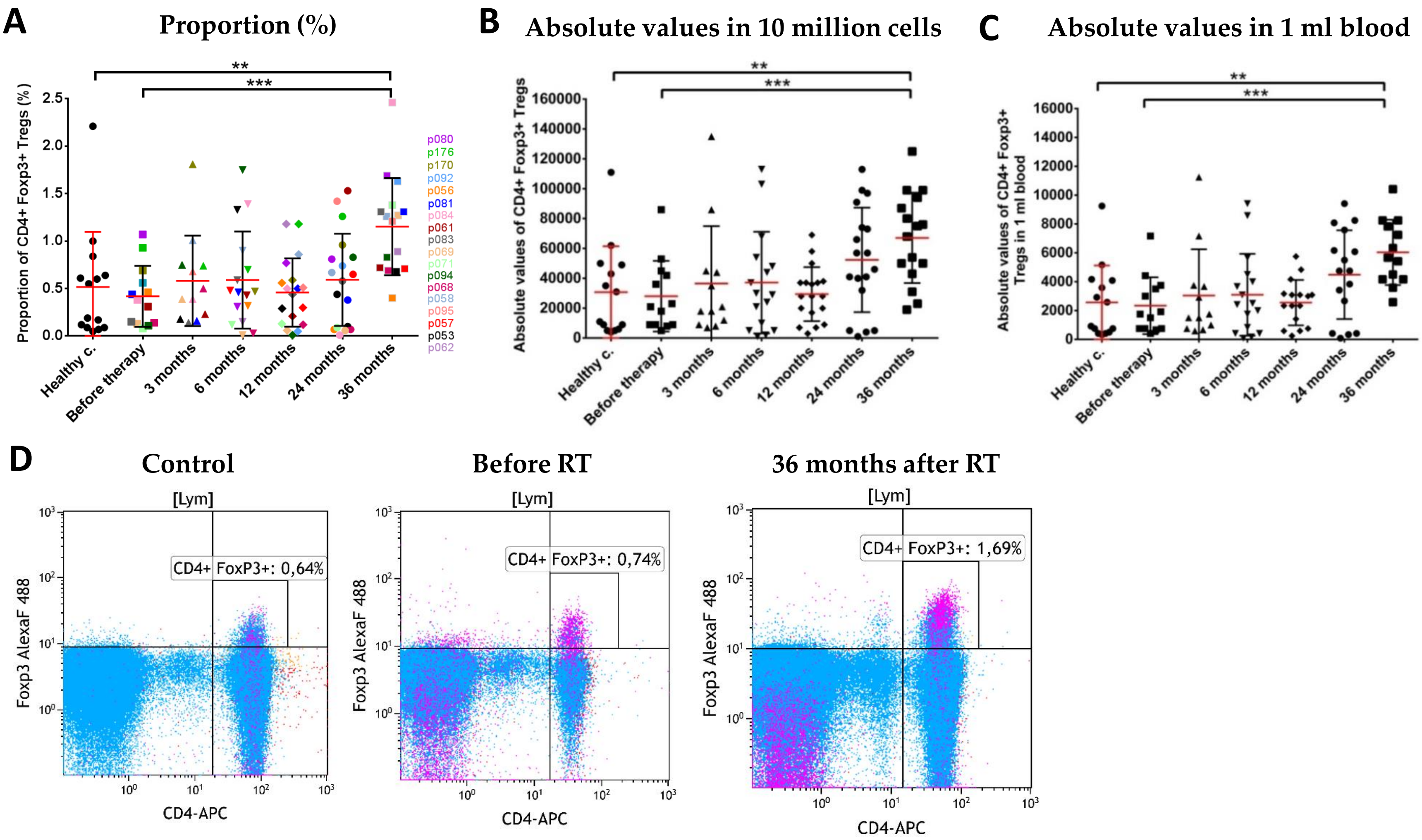

Supplementary Figure S4. Tregs increase in prostate cancer patients at late time point during the follow up. (A) Changes in the proportion of Tregs. To investigate individual changes spots representing individual patients were coloured differently. Absolute Treg values normalized either to 10 million cells (B) or to 1 ml blood (C). (D) Representative dot plots of Tregs of a healthy control and a prostate cancer patient before and 36 months after radiotherapy. Blood collection, processing and immune phenotyping was carried out as described in Materials and methods at the indicated time points relative to brachytherapy. *n* (healthy controls) = 14, *n* (cancer patients) = 7-18. Significant differences were indicated with * ($p < 0.05$) ** ($p < 0.01$) and *** ($p < 0.001$).

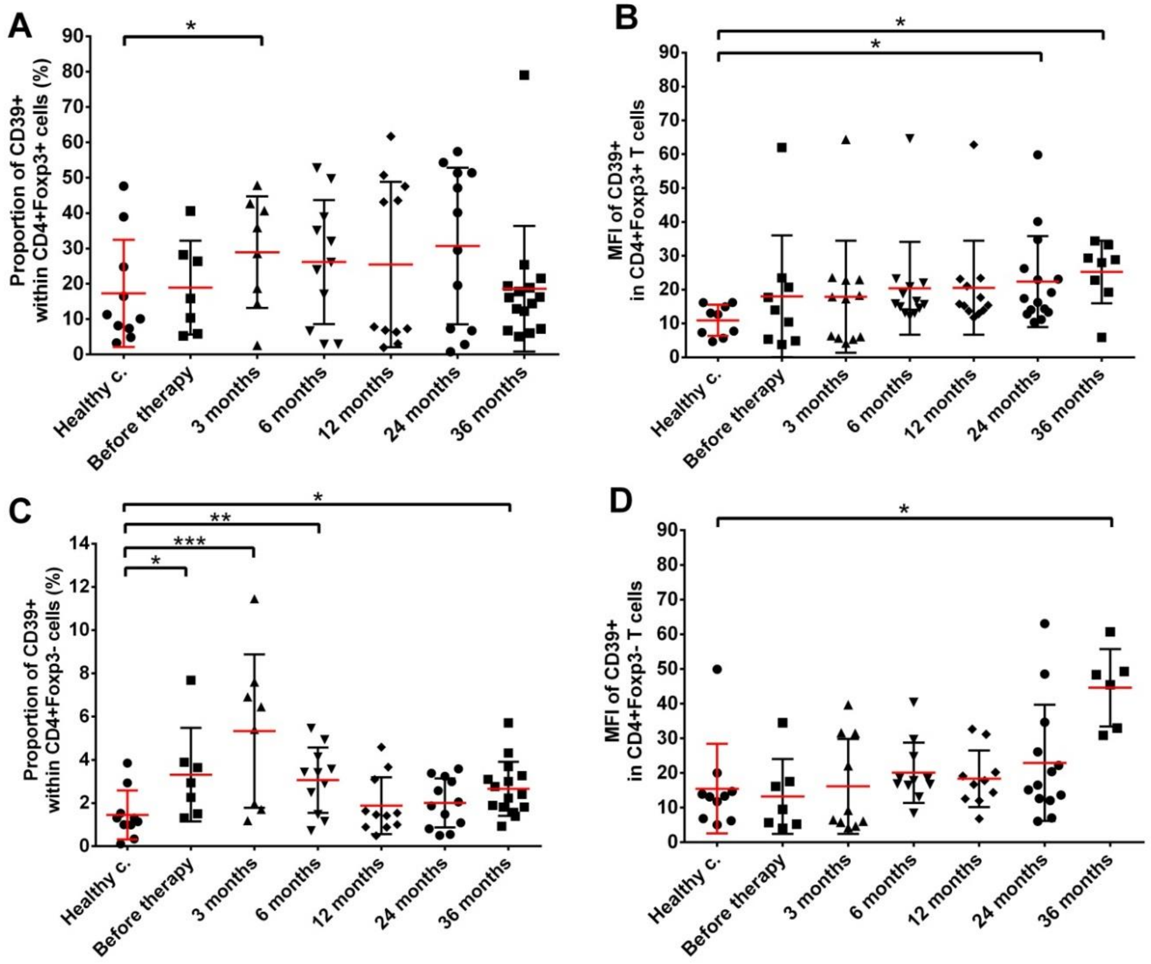

Supplementary Figure S5. CD39 expression increases on CD4+ effector T cells at late time point during the follow up. (A) Fraction of CD39 expressing Tregs; (B) CD39 expression level on Tregs; (C) fraction of CD39 expressing CD4+ Teff cells, (D) CD39 expression level on CD4+Foxp3- effector T cells as determined by MFI. Blood collection, processing and immune phenotyping was carried out as described in Materials and methods at the indicated time points relative to brachytherapy. *n* (healthy controls) = 10, *n* (cancer patients) = 7-18. Significant differences were indicated with * ($p < 0.05$) ** ($p < 0.01$) and *** ($p < 0.001$).

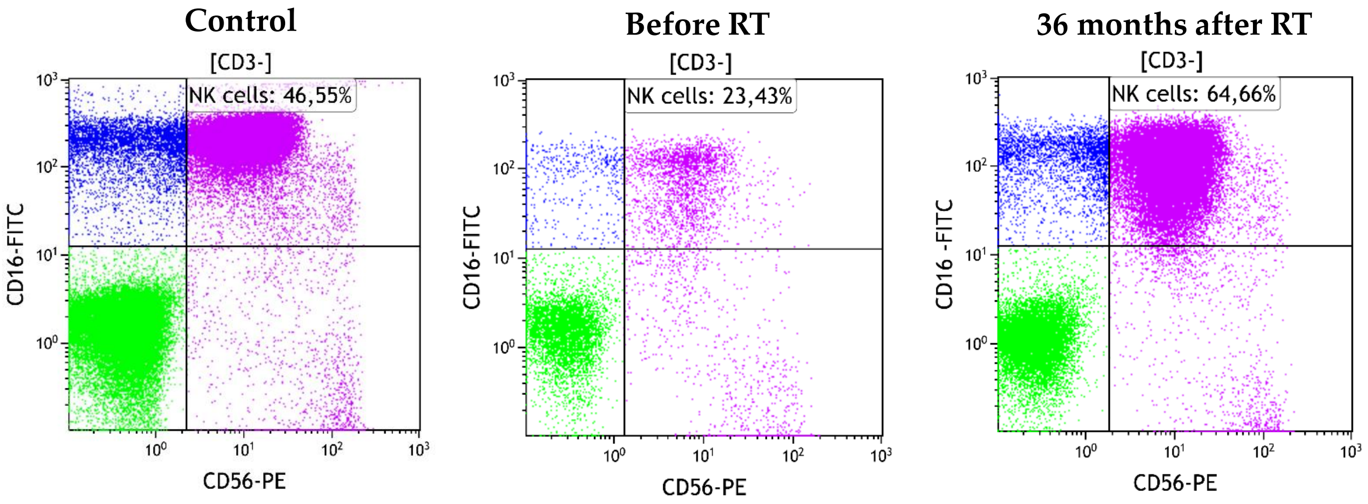

Supplementary Figure S6. Representative dot plots of NK cells are shown of a healthy control and a prostate cancer patient before and 36 months after radiotherapy.

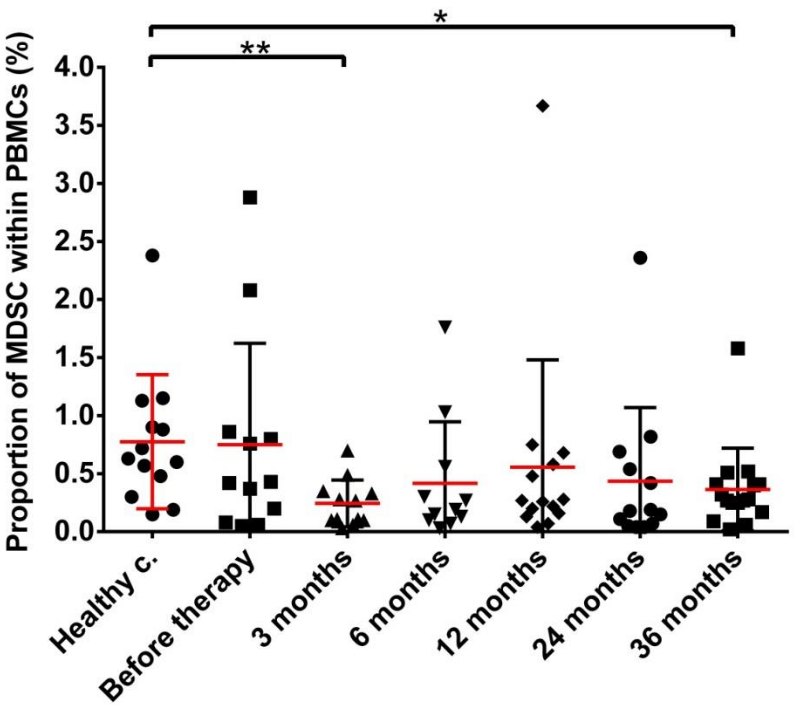

Supplementary Figure S7. MDSC levels decreased in prostate cancer patients after therapy. Blood collection, processing and immune phenotyping was carried out as described in Materials and methods at the indicated time points relative to brachytherapy. *n* (healthy controls) = 14, *n* (cancer patients) = 11-18. Significant differences were indicated with * ($p < 0.05$) and ** ($p < 0.01$).

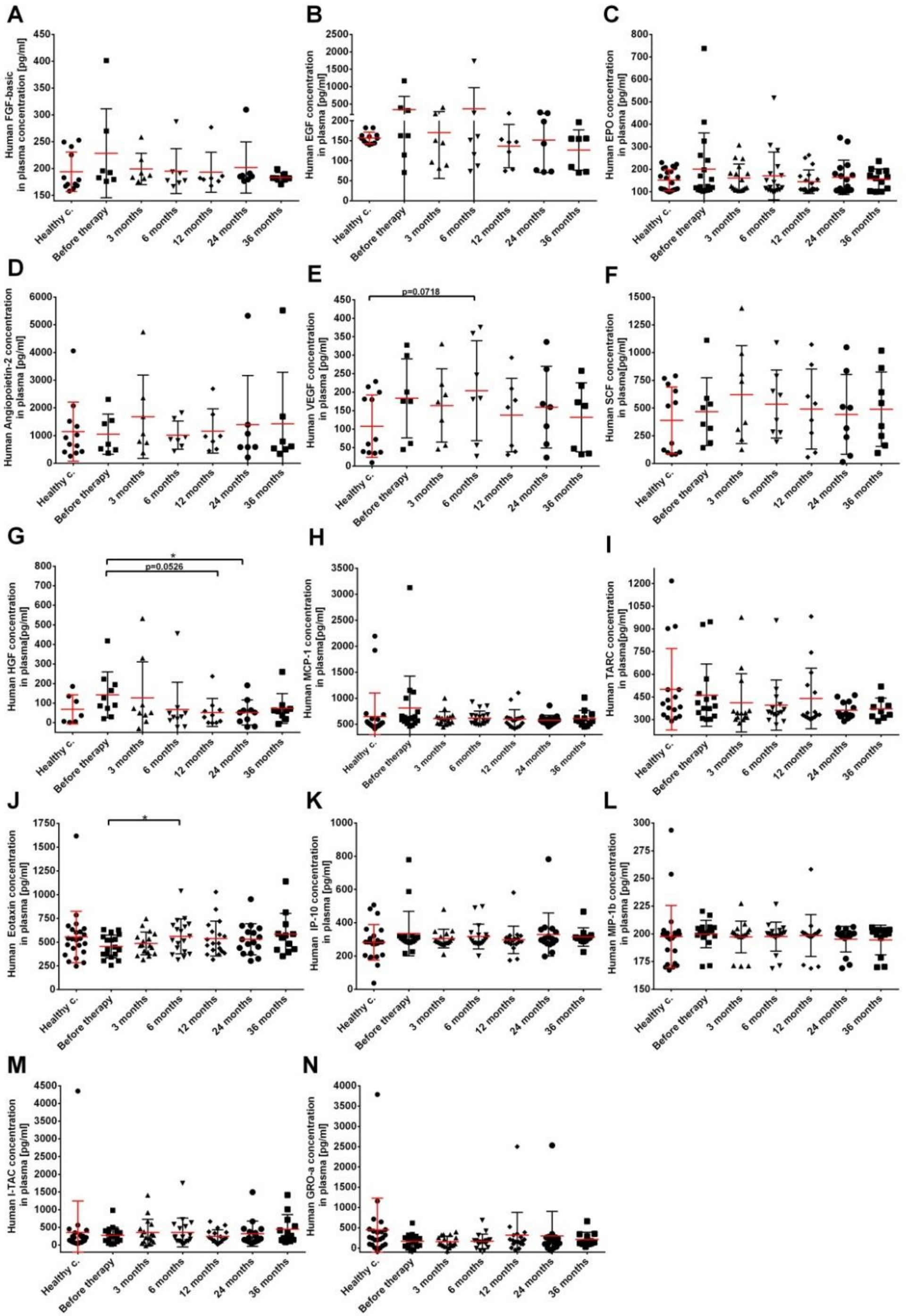

Supplementary Figure S8. No significant changes in plasma proteins (A: human FGF-basic, B: human EGF, C: human EPO, D: human Angiopoietin-2, E: human VEGF, F: human SCF, G: human HGF, H: human MCP-1, I: human TARC, J: human Eotaxin, K: human IP-10, L: human MIP-1b, M: human I-TAC, N: human GRO-α) were detected in prostate cancer patients compared to healthy controls. Blood collection, processing and plasma analysis was carried out as described in Materials and methods at the indicated time points relative to brachytherapy. *n* (healthy controls) = 12-24, *n* (cancer patients) = 12-18.

Supplementary Tables

Human growth factor panel	
**Protein name**	**Abbreviation**
**Angiopoietin-2**	Ang-2
**Epidermal growth factor**	EGF
**Erythropoietin**	EPO
**Fibroblast growth factor basic**	FGF-basic
**Granulocyte colony stimulating factor**	G-CSF
**Granulocyte-macrophage colony-stimulating factor**	GM-CSF
**Hepatocyte growth factor**	HGF
**Macrophage Colony-Stimulating Factor**	M-CSF
**Platelet Derived Growth Factor AA**	PDGF-AA
**Platelet Derived Growth Factor BB**	PDGF-BB
**Stem cell factor**	SCF
**Transforming growth factor-α**	TGF-α
**Vascular endothelial growth factor**	VEGF
**Human proinflammatory and chemokine panel**	
**Monocyte Chemoattractant Protein-1**	MCP-1/CCL2
**Regulated upon Activation, Normal T cell Expressed and Secreted**	RANTES/CCL5
**Interferon gamma-induced protein-10**	IP-10/CXCL10
**Eotaxin**	CCL11
**Thymus-and Activation-Regulated Chemokine**	TARC/CCL17
**Macrophage inflammatory protein-1α**	MIP-1α/CCL3
**Macrophage inflammatory protein-1β**	MIP-1β/CCL4
**Monokine induced by gamma interferon**	MIG/CXCL9
**Macrophage inflammatory protein-3α**	MIP-3α/CCL20
**Epithelial-derived neutrophil-activating peptide-78**	ENA-78/CXCL5
**Growth-regulated oncogene-α**	GROα/CXCL1
**Interferon-inducible T-cell Alpha Chemoattractant**	I-TAC/CXCL11
**Interleukine-8**	IL-8/CXCL8

Supplementary Table S1. The list of the investigated protein names and their abbreviations.

Patient code and subcode	Genitourinalis/Genitourinary (GU) side effects	Gastrointestinal (GI) side effects	Cumulative acute GU side effects	Cumulative acute GI side effects	Cumulative chronic GU side effects	Cumulative chronic GI side effects	International Prostate Symptom Score (IPSS)	Quality of life (Qol)
**P053/0**	-	-	-	-	-	-	5	2
**P053/3**	2	0	2	0	-	-	10	2
**P053/6**	2	0	-	-	2	0	10	2
**P053/12**	2	1	-	-	2	1	10	2
**P053/24**	0	0	-	-	2	1	8	2
**P053/36**	0	0	-	-	2	1	10	1
**P056/0**	-	-	-	-	-	-	-	-
**P056/3**	2	0	2	0	-	-	21	3
**P056/6**	2	0	-	-	2	0	23	2
**P056/12**	2	0	-	-	2	0	15	4
**P056/24**	2	0	-	-	2	0	28	4
**P056/36**	2	0	-	-	2	0	24	2
**P057/0**	-	-	-	-	-	-	3	1
**P057/3**	1	0	1	0	-	-	6	1
**P057/6**	0	0	-	-	0	0	2	0
**P057/12**	0	0	-	-	0	0	3	1
**P057/24**	0	0	-	-	0	0	2	1
**P057/36**	0	0	-	-	0	0	2	1
**P058/0**	-	-	-	-	-	-	4	0
**P058/3**	2	0	2	0	-	-	21	2
**P058/6**	2	0	-	-	2	0	12	2
**P058/12**	0	0	-	-	2	0	6	2
**P58/24**	0	0	-	-	2	0	4	1
**P058/36**	0	0	-	-	2	0	4	1
**P061/0**	-	-	-	-	-	-	1	1
**P061/3**	0	0	0	0	-	-	6	1
**P061/6**	0	0	-	-	0	0	2	0
**P061/12**	0	0	-	-	0	0	2	1
**P061/24**	0	0	-	-	0	0	2	1
**P061/36**	0	0	-	-	0	0	1	1
**P062/0**	-	-	-	-	-	-	14	4
**P062/3**	2	0	2	0	-	-	16	3
**P062/6**	2	0	-	-	2	0	10	4
**P062/12**	2	0	-	-	2	0	13	5
**P062/24**	2	2	-	-	2	2	20	5
**P062/36**	1	0	-	-	2	2	10	2
**P068/0**	-	-	-	-	-	-	-	-
**P068/3**	2	0	2	0	-	-	27	2
**P068/6**	1	0	-	-	1	0	12	1

**P068/12**	0	0	-	-	1	0	9	0
**P068/24**	0	0	-	-	1	0	4	0
**P068/36**	0	0	-	-	1	0	5	0
**P069/0**	-	-	-	-	-	-	-	-
**P069/3**	2	0	2	0	-	-	14	3
**P069/6**	2	1	-	-	2	1	9	3
**P069/12**	0	0	-	-	2	1	0	1
**P069/24**	1	0	-	-	2	1	4	1
**P069/36**	1	0	-	-	2	1	4	1
**P071/0**	-	-	-	-	-	-	-	-
**P071/3**	0	0	0	0	-	-	8	1
**P071/6**	0	0	-	-	0	0	3	1
**P071/12**	0	0	-	-	0	0	2	1
**P071/24**	0	0	-	-	0	0	2	1
**P071/36**	0	0	-	-	0	0	2	1
**P072/0**	-	-	-	-	-	-	-	-
**P072/3**	2	0	2	0	-	-	22	1
**P072/6**	2	0	-	-	2	0	15	4
**P080/0**	-	-	-	-	-	-	3	1
**P080/3**	2	0	2	0	-	-	22	2
**P080/6**	1	0	-	-	1	0	3	0
**P080/12**	0	0	-	-	1	0	2	0
**P080/24**	0	0	-	-	1	0	1	1
**P080/36**	0	0	-	-	1	0	1	0
**P081/0**	-	-	-	-	-	-	11	3
**P081/3**	2	0	2	0	-	-	19	4
**P081/6**	2	0	-	-	2	0	24	3
**P081/12**	2	0	-	-	2	0	22	2
**P081/24**	1	0	-	-	2	0	14	3
**P081/36**	1	0	-	-	2	0	14	3
**P083/0**	-	-	-	-	-	-	4	1
**P083/3**	2	0	2	0	-	-	19	4
**P083/6**	1	0	-	-	1	0	9	1
**P083/12**	0	0	-	-	1	0	2	1
**P083/24**	1	0	-	-	1	0	6	1
**P083/36**	0	0	-	-	1	0	8	1
**P084/0**	-	-	-	-	-	-	-	-
**P084/3**	2	0	2	0	-	-	13	1
**P084/6**	1	0	-	-	1	0	5	1
**P084/12**	0	0	-	-	1	0	2	1
**P084/24**	0	0	-	-	2	1	3	0
**P084/36**	0	0	-	-	1	0	5	0
**P085/0**	-	-	-	-	-	-	7	1
**P085/3**	2	1	2	1	-	-	7	2
**P085/6**	2	1	-	-	2	1	3	1
**P085/12**	0	0	-	-	2	1	11	2

**P091/0**	-	-	-	-	-	-	-	-
**P091/3**	2	0	2	0	-	-	17	3
**P091/6**	2	1	-	-	2	1	13	3
**P092/0**	-	-	-	-	-	-	10	1
**P092/3**	2	0	2	0	-	-	26	5
**P092/6**	2	0	-	-	2	0	17	3
**P092/12**	0	0	-	-	2	0	6	1
**P092/24**	0	0	-	-	2	0	3	1
**P092/36**	0	0	-	-	2	0	5	1
**P094/0**	-	-	-	-	-	-	4	1
**P094/3**	3	0	3	0	-	-	7	2
**P094/6**	1	0	-	-	1	0	6	1
**P094/12**	0	0	-	-	1	0	3	0
**P094/24**	0	0	-	-	1	0	3	0
**P094/36**	0	0	-	-	1	0	3	0
**P095/0**	-	-	-	-	-	-	3	0
**P095/3**	2	0	2	0	-	-	13	2
**P095/6**	2	0	-	-	2	0	8	2
**P095/12**	2	0	-	-	2	0	6	1
**P095/24**	-	-	-	-	2	0	2	0
**P094/36**	-	-	-	-	2	0	2	0
**P170/0**	-	-	-	-	-	-	7	1
**P170/3**	1	1	0	0	-	-	22	3
**P170/6**	1	1	-	-	1	1	9	1
**P170/12**	1	1	-	-	1	1	5	1
**P170/24**	0	1	-	-	1	1	9	2
**P176/0**	-	-	-	-	-	-	12	3
**P176/3**	1	0	1	0	-	-	11	3
**P176/6**	1	0	-	-	2	0	9	2
**P176/12**	2	0	-	-	2	0	10	3
**P176/24**	1	0	-	-	2	0	13	4

Supplementary Table S2. Side effects of prostate cancer patients receiving low-dose rate brachytherapy. The RTOG-EORTC radiation toxicity grading system is from 0 to 5; 0: patients do not have any side effects, 1: patients have symptoms but intervention is not required, 2: patients need medication, 3: diarrhea requiring parenteral support, severe mucous or blood discharge needing sanitary pads, abdominal distention, 4: critical state, 5: death directly related to radiation effects (Simone et al., 2008). According to IPSS the symptoms can be mild (symptom score ≤ 7), moderate (symptom score range 8-19) and severe (symptom score range 20-35) based on The American Urological Association (AUA) Symptom Index (Barry et al., 1992). The scores of Quality of Life due to urinary symptoms are: 0: delighted, 1: pleased, 2: mostly satisfied, 3: mixed, 4: mostly dissatisfied, 5: unhappy, 6: terrible. -: data not available

Patient subcoding: 0: before treatment, 3: 3 months after treatment, 6: 6 months after treatment, 12: 12 months after treatment, 24: 24 months after treatment, 36: 36 months after treatment.
